# New twinning route in face-centered cubic nanocrystalline metals

**DOI:** 10.1038/s41467-017-02393-4

**Published:** 2017-12-15

**Authors:** Lihua Wang, Pengfei Guan, Jiao Teng, Pan Liu, Dengke Chen, Weiyu Xie, Deli Kong, Shengbai Zhang, Ting Zhu, Ze Zhang, Evan Ma, Mingwei Chen, Xiaodong Han

**Affiliations:** 1 Institute of Microstructure and Property of Advanced Materials, Beijing Key Lab of Microstructure and Property of Advanced Materials, Beijing University of Technology, Beijing, 100124 China; 2 Materials Engineering, University of Queensland, Brisbane, QLD 4072 Australia; 3 Beijing Computational Science Research Center, Beijing, 100193 China; 4 WPI Advanced Institute for Materials Research, Tohoku University, Sendai, 980-8577 Japan; 5 Department of Material Physics and Chemistry, University of Science and Technology Beijing, Beijing, 100083 China; 6 Woodruff School of Mechanical Engineering, Georgia Institute of Technology, Atlanta, GA 30332 USA; 7 Department of Physics, Applied Physics, & Astronomy, Rensselaer Polytechnic Institute, Troy, New York, NY 12180 USA; 8 State Key Laboratory of Silicon Materials, Zhejiang University, Hangzhou, 310008 China; 9 Department of Materials Science and Engineering, Johns Hopkins University, Baltimore, MD 21218 USA

## Abstract

Twin nucleation in a face-centered cubic crystal is believed to be accomplished through the formation of twinning partial dislocations on consecutive atomic planes. Twinning should thus be highly unfavorable in face-centered cubic metals with high twin-fault energy barriers, such as Al, Ni, and Pt, but instead is often observed. Here, we report an in situ atomic-scale observation of twin nucleation in nanocrystalline Pt. Unlike the classical twinning route, deformation twinning initiated through the formation of two stacking faults separated by a single atomic layer, and proceeded with the emission of a partial dislocation in between these two stacking faults. Through this route, a three-layer twin was nucleated without a mandatory layer-by-layer twinning process. This route is facilitated by grain boundaries, abundant in nanocrystalline metals, that promote the nucleation of separated but closely spaced partial dislocations, thus enabling an effective bypassing of the high twin-fault energy barrier.

## Abstract (web summary)

Twin nucleation in face-centered cubic metals with high twin-fault energies should theoretically be unfavourable, but instead twinning is very often observed. Here, the authors report a new twinning route in nanocrystalline platinum that bypasses the high twin-fault energy barrier using closely spaced partial dislocations.

## Introduction

Twinning plays an important role in the plasticity and strengthening of metals and alloys^1–14^. It also provides possible solutions for enhancing the ductility of structural alloys in high-temperature applications^15^. In the classical twinning theory for face-centered cubic (FCC) metals, a deformation twin is nucleated through layer-by-layer movement of partial dislocations on consecutive, close-packed atomic planes^16–19^ via the pole mechanism^16^, prismatic gliding^17^, faulted dipoles^18^, stimulated slip, and other mechanisms^2,3,19–22^. In nanocrystalline (NC) metals, grain boundaries (GBs) can lead to twinning processes that are different from those of coarse-grained counterparts, and many alternative twinning routes, such as GB splitting, heterogeneous and homogeneous twin formation, have been proposed^4,21,23^. However, it is commonly believed that the classical pathway of twin nucleation should be preserved, i.e., a deformation twin must inevitably be nucleated through the layer-by-layer emission of partial dislocations on consecutive atomic planes without interruption^1–8,16–27^. According to this understanding, deformation twinning should be suppressed in many FCC metals with high unstable twin-fault energy (γ_utf_), such as Al, Ni, Pt, and Pd, in which the formation of twin embryos is difficult^7,8,28,29^. In addition, it has been recognized that the grain-size effect can lead to a transition of the deformation mechanism from full dislocations to partial dislocations in FCC NC metals^2,4,30^. The active partial dislocations can increase the likelihood of twinning in NC metals. However, in the theoretical analysis of twinning energy landscape, the initiation of twinning via formation of a layer-by-layer stacking fault (SF) configuration depends not only on the unstable SF energy (γ_usf_) but also, more decisively, on an energy barrier, known as the γ_utf_, and accordingly on the ratio of γ_utf_/γ_usf_ (see refs. ^5,6^). Twinning is, therefore, highly unfavorable in many metals such as Al, Ni, Pt, and Pd, because of their high γ_utf_. However, this theoretical prediction is at odds with an extensive body of experimental observations of deformation twins in these FCC NC metals^2,4,8^.

In this work, we utilized a recently developed technique of nanomechanical testing^31^ together with a spherical-aberration-corrected transmission electron microscope (Cs-corrected TEM) to capture the atomic-scale twinning process in NC Pt. Our in situ observations provide compelling evidence of a new route of deformation twinning in NC Pt having a high twin-fault energy barrier. This new twinning route features the emission of a partial dislocation between two previously formed SFs separated by one atomic layer, which contrasts with the classical twinning model of layer-by-layer formation of SFs. The new twinning pathway has a much lower energy barrier than the conventional layer-by-layer twinning. Our results thus provide a new mechanistic understanding on the formation of widely observed deformation twins in FCC NC metals with high twinning energy barriers.

## Results

### In situ atomic-scale observation of twin nucleation

The Pt thin film samples used in this work consist of nano-sized grains. Grain sizes range from 4 to 20 nm with an average value of 10 nm, and most grain sizes are <15 nm^30^. The in situ atomic-scale tensile experiments were performed with atomically resolved mechanical microscopy (ARMM), which integrates a nanomechanical testing device (see Supplementary Figs. 1–4 and Method) into a Cs-corrected TEM^32^.

**Fig. 1:**
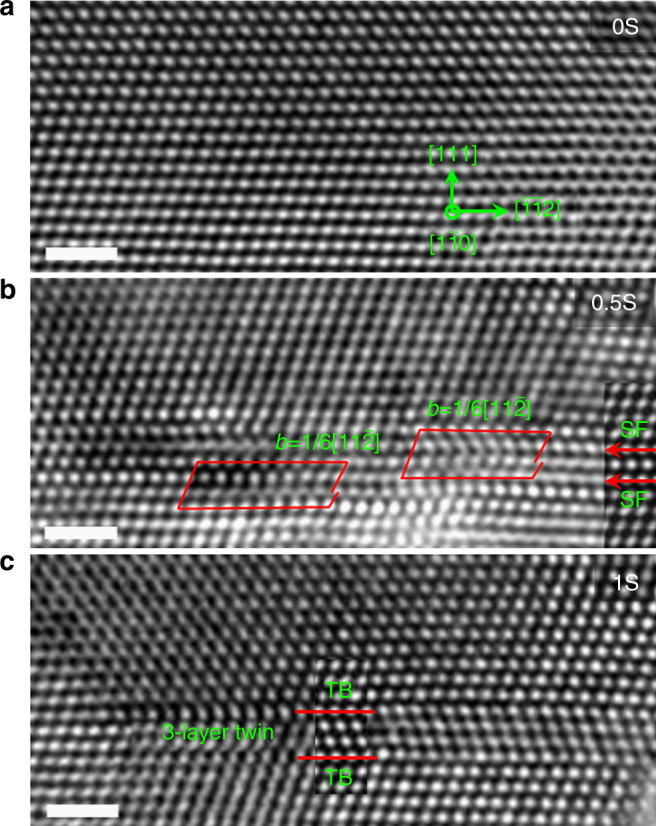
Aberration-corrected HRTEM images showing the nucleation of a three-layer twin. **a** No defects were observed initially. **b** Two stacking faults (SFs) separated by one atomic layer were observed during straining. **c** A three-layer twin was formed through the nucleation of a partial dislocation between the two previously formed SFs. Corresponding simulated HRTEM images are presented (using superimposed inserts) in **b**, **c**. It should be noted that white dots in HRTEM images may not directly correspond to atomic columns for a high atomic number material such as Pt. The scale bars are for 1 nm

Figure 1 shows the high-resolution TEM (HRTEM) images of a twin nucleation process, viewed along the $\left[ {1\bar 10} \right]$ direction and acquired 0.5 s apart during in situ tensile testing. Figure 1a is an HRTEM image taken prior to the elastic limit and hence no dislocations were observed. Further loading led to the formation of two SFs separated by a single atomic layer, which are referred to as 1–3 SFs on the basis of the relative positions of the 1st and 3rd atomic layers involved (Fig. 1b). These two SFs resulted from the emission of two partial dislocations from a GB; namely, a partial dislocation was emitted from the GB and then glided into the grain, leaving behind a SF. The Burgers vectors of the two partial dislocations are $\frac{1}{6}\left[ {11\bar 2} \right]$. The configuration of 1–3 SFs is confirmed by the HRTEM image simulation (Fig. 1b). With further straining, one more SF formed in between 1–3 SFs through the emission of a partial dislocation from the GB as shown in Fig. 1c (see similar examples of partial dislocation emission from GBs in Supplementary Figs. 5–9). Consequently, a three-layer twin nucleated, which is denoted as 1–3–2 SFs so as to highlight the sequence of the atomic layers involved during a twin nucleation process. The simulated HRTEM image of the three-layer twin is shown as an inset of Fig. 1c, which is consistent with the experimental HRTEM image. This process of twin nucleation is intrinsically different from the previous twinning models^1–8,16–27^ in which a twin embryo forms through the successive emission of partial dislocations on consecutive (111) planes. We note that GBs in NC Pt consist of a high density of atomic-sized steps that provide abundant and effective sources for SF nucleation. Cs-corrected HRTEM images (Supplementary Figs. 10–17) show the GB structures and the statistics of GB step sizes. It is seen that the most frequently observed GB steps in nanograins with sizes in between 6 and 10 nm are three atomic spacings in width, i.e., the step edges are three atomic layers apart. As revealed in experiments (Supplementary Fig. 15) and molecular dynamics (MD) simulations (Supplementary Figs. 18–20), such steps are the sources where the close-neighbored 1–3 SFs emerge.

A peak-pair analysis (PPA)^33,34^ was performed to quantify the local shear strains during loading, from which the local shear stresses of twin nucleation were estimated. A large unstrained area was used as the reference for strain mapping. Using this method, the local elastic shear strain near the partial dislocation nucleation zone was directly mapped (Supplementary Fig. 21). The shear strain maps (along the $\left[ {11\bar 2} \right]$ direction) of *ε*_*xy*_ indicated that the maximum shear strain was ~2.2% for the nucleation of 1–3 SFs. The shear strain for emission of a partial dislocation in between 1–3 SFs remained at the same level. Thus, the shear stresses for partial dislocation emission and accordingly twin nucleation were estimated to be ~1.44 GPa for Pt with a shear modulus of 65.4 GPa^8^.

**Fig. 2:**
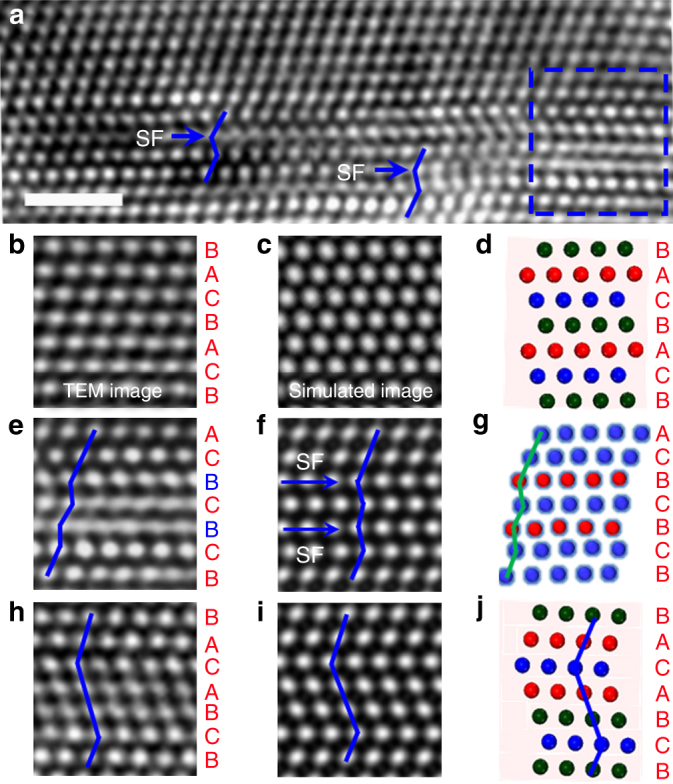
HRTEM images and associated schematic illustrations showing the nucleation of a three-layer twin. **a** An enlarged HRTEM image corresponding to Fig. 1b. **b** A local region in **a** showing the perfect FCC lattice structure. **c** Simulated HRTEM image corresponding to **b**. **d** Schematic of the ABCABC stacking sequence in an FCC lattice. **e** An enlarged view of the boxed region in **a**. **f** Simulated HRTEM image corresponding to **e**. **g** Schematic of two SFs separated by one atomic layer (i.e., 1–3 SFs), as derived from the HRTEM image in **e**. The atomic layer with red atoms indicates the SF. **h** An enlarged HRTEM image showing a three-layer twin. **i** Simulated HRTEM image corresponding to **h**. **j** Schematic illustration of three-layer twin formed by nucleation of a SF in between 1–3 SFs. The scale bars are for 1 nm

**Fig. 3:**
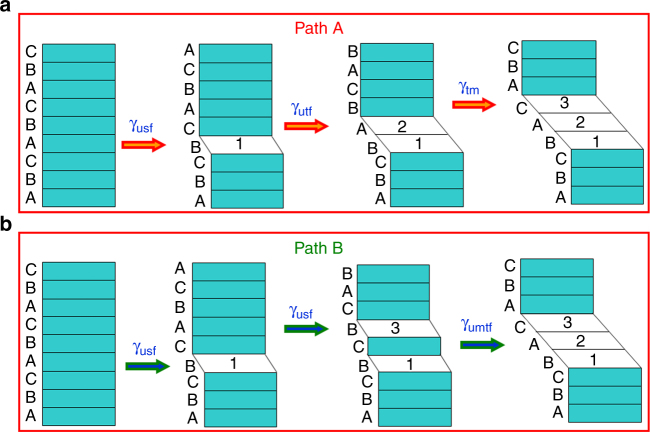
Schematic illustration of the traditional and new twin nucleation routes. **a** In the traditional twinning route (path A), twin nucleation involves the sequential nucleation of three partial dislocations and accordingly three SFs on consecutive atomic layers. The highest energy barrier for path A is the unstable twin-fault energy γ_utf_. **b** In the new twinning route (path B), two partial dislocations (i.e., two SFs) are present, but they are separated with one atomic layer in between; then a third partial dislocation forms in between the two previously formed SFs, resulting in a three-layer twin. The highest energy barrier becomes the unstable middle-twin-fault energy γ_umtf_

Figure 2a shows an enlarged view of the HRTEM image presented in Fig. 1b. For comparison, Fig. 2b shows the image of a defect-free region in which three consecutive (111) planes, labeled as A, B, and C, form a repeating unit. The simulated HRTEM image and the corresponding schematic illustration are presented in Fig. 2c, d, respectively. After the formation of 1–3 SFs, the stacking sequence is changed from ABCABCABCA in an FCC lattice to ABCBCBCABC containing a local hexagonal close-packed (HCP) lattice, as shown in Fig. 2e. According to the atomic model (see Fig. 3 below), the simulated HRTEM image (Fig. 2f) is consistent with the enlarged HRTEM image (Fig. 2e) corresponding to the boxed region in Fig. 2a. The 1–3 SFs in Fig. 2f are indicated by blue arrows. A schematic illustration of the atomic stacking sequence derived from atomic positions in Fig. 2e is shown in Fig. 2g, where the red atomic layers represent 1–3 SFs. After the emission of a $\frac{1}{6}\left[ {11\bar 2} \right]$ partial dislocation on the middle plane between 1–3 SFs, the stacking sequence of ABCBCBCABC is changed to ABCBACABCA, where the local lattice with a BAC stacking sequence exhibits a twin-symmetry relationship with the matrix (Fig. 2h–j). Such a twin nucleation pathway with 1–3–2 SFs was observed visually more than ten times during the in situ TEM experiment. Some of these events proceeded over a sufficiently long period of time (~seconds), allowing images to be taken, as recorded in Supplementary Figs. 5–9. This indicates the new twinning route is not a rare event.

### The classical and new twinning models

**Fig. 4:**
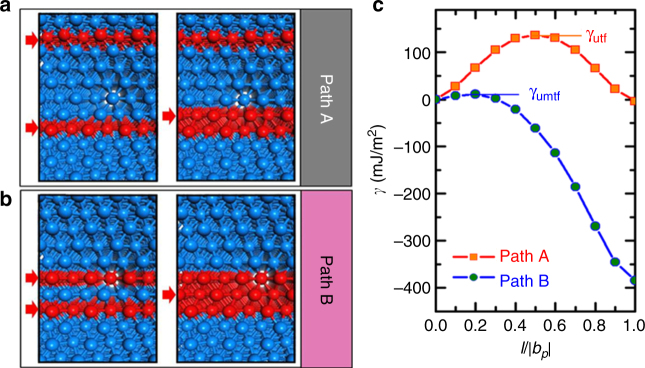
Density functional theory calculations of twinning energy landscape. **a** Atomic structures of twin nucleation in the classical model (path A). In the initial state (the left image), the first two SFs are separated by five atomic layers; in the final state (the right image), a third SF forms next to a SF. **b** Atomic structures of twin nucleation in the new model (path B). In the initial state (the left image), the first two SFs are separated by one atomic layer; in the final state (the right image), a third SF forms between the first two SFs. In **a**, **b**, the red atomic planes indicated the SF layers. **c** The calculated energy change *γ* as a function of the sliding displacement *l* (normalized by the magnitude of twin partial slip *b*_p_) at the SF layer for paths A and B. The energies of the initial states in **a**, **b** are set to zero

Figure 3 shows a schematic illustration for both the classical twinning model (referred to as path A) and the new twinning model observed in our experiments (referred to as path B). The green color indicates the FCC configuration, while the white color the sheared plane. For path A (Fig. 3a), the formation of three SFs on consecutive layers changes the original FCC lattice of ABCABCABCABCABCA to ABCABC**B**CABCABCAB, ABCABC**BA**BCABCABC, and ABCABC**BAC**ABCABCA sequentially. As marked by the arrows, the formation of the first SF changes the local FCC structure into the HCP one, which is strictly an incipient HCP structure due to its one-atom thickness. The subsequent formation of the second and third SF occurs based on this HCP structure. Because of the strong interaction between neighboring SFs, each step of SF formation experiences a similarly high twin-fault energy barrier of γ_utf_ (Supplementary Fig. 22 and 23). For path B (Fig. 3b), the formation of 1–3 SFs involves the emissions of two separated partial dislocations. This would also encounter a high SF energy barrier of γ_usf_. However, such emissions are initiated from, and greatly assisted by, GB steps which usually have sizes of a few atomic layers in NC metals and alloys^2,4,23,30^. In our experiments, the GB steps of three atomic-layers wide were most commonly observed in grains with a size of ~6–10 nm (Supplementary Fig. 10). These steps facilitated the nucleation of 1–3 SFs and accordingly 1–3–2 twins during plastic deformation. Sometimes, we also observed the GB steps of four or five atomic layers thick in those grains (Supplementary Figs. 13–15). At those steps, SFs were usually separated by four or more atomic layers, such that they could not develop into twins. In contrast, in grains larger than ~10 nm, the GB steps with four and more layers were frequently observed (Supplementary Fig. 12). However, full dislocations instead of twins were commonly observed in those grains (Supplementary Fig. 16). On the other hand, the GB steps of one or two layers thick were more commonly seen in grains smaller than ~6 nm (Supplementary Fig. 11). In those grains, the GB-mediated processes such as grain rotation dominated (Supplementary Fig. 17) and twin nucleation was rarely observed. Importantly, the 1–3 SFs form a local high-energy HCP structure embedded in a low-energy FCC matrix. Subsequent emission of a partial dislocation between the 1–3 SFs returns the local high-energy HCP structure to the low-energy FCC structure and thus experiences a much lower energy barrier. The third partial dislocation emission also releases the elastic strains around the 1–3 SFs, which markedly reduces the local strain energy. This favorable twinning process is confirmed by our DFT calculations (Fig. 4 and Supplementary Fig. 24) and MD simulation (Supplementary Figs. 18–20). The latter also shows the frequent formation of 1–3 SFs from GBs containing a high density of atomic-scale steps, which agrees with the experimental observations shown in Figs. 1 and 2 and Supplementary Figs. 5–9. It is noteworthy that the formation of 1–3–2 twins was more frequently observed in Cu than in Pt during our MD simulations. This further confirms that the GB geometry from the nano-size effect plays an important role in 1–3–2 twinning even in FCC metals with lower twin-fault energy.

### Ab initio modeling of twin formation

Based on the twinning models in Fig. 3, the energy landscape underlying the new twinning route was evaluated using ab initio density functional theory (DFT) calculations. We constructed a reference model system for path A, representing the classical layer-by-layer twinning route. In this model, we randomly inserted the first and second SFs separated by five atomic layers (see the left image in Fig. 4a). On the other hand, considering that a high density of isolated SFs (or equivalently partial dislocations) was frequently observed in NC Pt samples during the early stage of plastic deformation (Supplementary Figs. 5–9 and Supplementary Figs. 13–15), we also constructed a model system for path B, representing the new twinning route revealed by our HRTEM observations. In this model, 1–3 SFs were introduced with one atomic layer in between (see the left image in Fig. 4b). Based on DFT results of energy and charge density (Supplementary Figs. 22–24), we found that the total energies of both systems are nearly identical. However, the energy barriers of twin nucleation are markedly different between the two paths. For path A, the next step of twin nucleation involves the formation of a partial dislocation on the (111) plane adjacent to the previously formed SF (see the right image in Fig. 4a), which must overcome a barrier of γ_utf_. In contrast, for path B, twin nucleation proceeds by the emission of a partial dislocation between 1–3 SFs (see the right image in Fig. 4b). The energy landscapes for paths A and B from DFT calculations are shown in Fig. 4c. The energy barrier for path A is associated with the classical unstable twin-fault energy γ_utf_ and is approximately 124 mJ/m^2^ higher than the unstable middle-twin-fault energy (denoted as γ_umtf_) for path B, which is only approximately 11 mJ/m^2^. As shown in Supplementary Fig. 24, our DFT calculations indicate that Al also has a relatively high value of γ_utf_ compared to γ_umtf_, similar to Pt. In contrast to Pt and Al, Cu has similar barriers for path A and B; this suggests both twinning routes could be taken for NC Cu. These results explain why deformation twins are frequently observed in experiments but highly unfavorable in the classical models of 1–2–3 twinning for FCC metals such as Al, Ni, Pt, and Pd^2,4,8^.

## Discussion

According to the classical twinning models, the probability of twin formation in FCC metals is determined by the ratio of γ_utf_/γ_usf_, and deformation twinning should not occur in metals with high γ_utf_/γ_usf_ ratios, such as Al, Ni, Pt, and Pd^28,29,35–38^. Our results indicate that the lower twinning energy barrier encountered along path B allows the high twin-fault energy barrier in NC Pt to be bypassed, thereby significantly increasing the possibility of twinning in NC Pt (Supplementary Fig. 24). For NC metals with high γ_utf_/γ_usf_ ratios but low γ_umtf_, such as Pt, our HRTEM observations and DFT calculations demonstrate that the mechanism of deformation twinning is dominated by path B, which has a significantly lower barrier compared to path A (Fig. 4, Supplementary Fig. 24). This finding is consistent with our related experimental observations; namely, extrinsic SFs (i.e., 1–2 SFs, regarded as twin embryos formed through the emission of two partial dislocations on neighboring (111) planes) as well as twin nucleation via path A are rarely observed. However, for NC metals with relatively low twin-fault energy barriers γ_utf_ (i.e., γ_utf_/γ_usf_ ≈ 1), such as Ag, Au, and Cu^7,28,29,37,38^, it is possible that both paths A and B are selected, as there is no significant difference between γ_utf_ and γ_umtf_ (Supplementary Fig. 24d). Once a twin has nucleated (from 0 to 3 layers), its subsequent growth could proceed more easily because of the relatively low-energy barrier for twin boundary migration^7,29,37,38^. This is supported by our in situ atomic-scale observations (Supplementary Fig. 25) as well as by previous calculations and MD simulations^7,13,29,37,38^.

It should be noted that partial dislocations are a prerequisite for twin nucleation. In coarse-grained metals with high γ_utf_/γ_usf_ ratios, deformation twins cannot predominantly nucleate under typical laboratory loading conditions, even though path B is energetically favorable. This is because plastic deformation in this case is dominated by full dislocations^2,5,7,8,30^. For nano-sized grains, however, the size effect leads to a transition of the deformation mode from full to partial dislocations^2,4,30,35^. Therefore, the likelihood of twinning becomes sufficiently high, such that deformation twins are frequently observed in NC materials. However, according to previous theoretic predictions, twinnability is a function of generalized SF energies and twinning is not favored in FCC metals of Pt, Al, and Ni^39,40^. That is, if twin nucleation were to follow path A, twinning would be unfavorable in these metals because of the high-energy barrier γ_utf_, and thus, only SFs resulting from partial dislocations would be observed. With the new 1–3–2 route, i.e., path B, which can effectively bypass the high twinning energy barrier, the nucleation of deformation twins in NC metals with high γ_utf_/γ_usf_ ratios becomes favored.

Compared with the classical twinning mechanisms in perfect crystals and coarse-grained FCC materials^16–22^, the twinning mechanisms in FCC NC materials are more complex, and partial dislocations are typically emitted from GBs^8^. Previous experimental studies and MD simulations suggest that deformation twins can nucleate in NC Al through dissociation and migration of GBs^2,8,21,41–44^. Atomistic simulations also suggest that deformation twinning in NC Al depends on the texture of NC samples^8,21,45^ and on the relative orientation between grains and the tensile loading direction^41^. In addition, dislocation reactions and cross-slip mechanisms^4,44^ as well as multiple dislocation sources^4,23^ have been proposed as possible origins of twinning activities in NC Ni and Cu^4^. Nevertheless, all of these mechanisms of twin nucleation for FCC NC materials are explained by the classical twinning models involving the layer-by-layer formation of twin faults^16–22^, and, however, do not resolve the concerns from the analysis of twinning energy landscape^2,4–8,28,29,46^. Our experiments show that for metals with high γ_utf_/γ_usf_ ratios and low γ_umtf_, a new twin nucleation process can proceed through the formation of 1–3 SFs and followed by emission of a partial dislocation in between. This route can effectively bypass the high twinning-fault energy barrier γ_utf_, as supported by our DFT calculations (Fig. 4 and Supplementary Figs. 22–24).

In conclusion, we report a twinning route in NC Pt on the basis of in situ atomic-scale observations and DFT calculations as well as MD simulations. In the presence of copious GB steps in NC metals, the closely spaced SFs form and thus facilitate a twinning route that has a much lower energy barrier than that the classical one. The finding of this twinning route explains the widely observed occurrence of deformation twins in FCC NC metals with high twinning energy barriers, such as Pt and Al as well as Ni and Pd. This study thus offers atomic-level insights into the plastic deformation behavior of FCC NC metals.

## Methods

### In situ TEM tensile experiment

The TEM tensile device consisted of two thermally actuated bimetallic strips, which were fixed on a TEM Cu-ring grid using superglue or epoxy resin (Supplementary Fig. 1a). Each bimetallic strip was composed of two layers of different materials with a large mismatch in thermal expansion coefficient, which facilitates the generation of large deflections at relatively low operating temperatures. Under an optical microscope, the bimetallic strips were well aligned to be vertical to the thin film samples. The thin films were attached to the surfaces of the bimetallic strips by epoxy resin (Supplementary Fig. 1b). Upon etching away the silica layer, the free-standing bimetallic strips together with the thin film tensile samples were released from the silicon substrate (Supplementary Fig. 1c). Then the bimetallic strips with the thin films attached were loaded onto a conventional TEM heating stage, which was operated as a double-tilt, displacement-controlled deformation stage. During subsequent TEM observations, the temperature controller could accurately increase the temperature of the double-tilt TEM tensile stage, so as to deform the bimetallic strips for exerting uniaxial tensile forces on the thin films (Supplementary Fig. 1d). The bimetallic strips have the length of ~2.2 mm, and the distance between the two bimetallic strips is ~20 µm. Under gentle heating, outward deflection of each bimetallic strip can be achieved to a maximum of <2 µm (according to our in situ TEM measurement), which is only 0.091% of the bimetallic strip length (Supplementary Fig. 2). Thus, the uniaxial tensile test of the Pt thin film can be performed, and the local and global strains can be measured from the TEM images during loading (Supplementary Figs. 3 and 4).

Using the above method, the strained grains were oriented in a low-index crystallographic orientation for HRTEM imaging, such that the atomic-scale deformation process was recorded during loading. The real-time evolution of microstructures in the thin film sample was recorded using a JEM-2100F TEM equipped with two Cs-correctors for both the imaging and probe-forming lens systems. The corrector was optimized for HRTEM imaging with a point-to-point resolution of 0.8 Å operated at 200 kV. The tensile tests were conducted at a temperature below 80 °C, which is much lower than the melting temperature (1772 °C) of Pt. Thus, the temperature effect on dislocation and twin nucleation could be neglected during the loading process. The atomic models of the HRTEM image in Fig. 2 were created using the commercial software CrystalKit. Based on these models, we performed the HRTEM simulations using Mactempas with the Multislice method. The parameters for the image simulation were as follows: zone axis, $\left[ {1\bar 10} \right]$; sample thickness, 10 nm; accelerating voltage, 200 kV; spherical-aberration coefficient, 1 µm; focus spread, 80 Å; defocus, 9 nm (over focus).

### Modeling

We performed ab initio calculations using the Vienna Ab initio Simulation Package based on DFT with a plane-wave, pseudopotential formalism^47^. Exchange and correlation effects were included within the Perdew-Burke-Ernzerhof form of the generalized gradient approximation^48,49^. For bulk FCC Pt, a 20 × 20 × 20 Monkhorst–Pack grid was used to integrate over the Brillouin zone of the elemental FCC unit cell, and the equilibrium lattice constant of FCC Pt was calculated to be 3.91 Å, in good agreement with that determined by experiment (3.92 Å). To investigate the energy barriers for deformation along the $\left[ {11\bar 2} \right]$ direction of FCC Pt, we used a supercell geometry consisting of a twelve-layer {111} slab with a $\surd 6 \times \surd 2$ surface cell (i.e., eight atoms per layer), separated by a vacuum gap equivalent to a further six layers. To obtain the energy barrier of each twin-forming pathway, calculations were performed using a plane-wave cutoff energy of 500 eV and 3 × 5 **k** points in the surface Brillouin zone. The total energy convergence criterion was 0.01 meV. All atoms in the system were allowed to relax in the <111> direction until the largest residual force on each atom was less than 0.02 ÅeV. A thermal broadening of 0.02 eV was applied to improve the convergence of the self-consistent procedure, and non-spin polarization was employed in all calculations.

In addition, we performed MD simulations using the interatomic potential of Pt^50^ as well as Cu^51^ for studying the 1–3–2 twinning processes at GBs. For example, Supplementary Fig. 18 shows a representative MD result in a Pt bi-crystal with a symmetric ${\sum} {\mathrm{9}}$ tilt GB containing a high density of atomic-sized steps. The simulation supercell has a total of 31008 atoms and is subjected to periodic boundary conditions. A uniaxial tensile stress was applied perpendicular to the GB, causing the frequent emissions of 1–3 SFs from GB steps.

### Data availability

The data that support the findings of this study are available from the corresponding author upon reasonable request.

## Electronic supplementary material

Supplementary Information
