# Supplementary material for: New twinning route in face-centered cubic nanocrystalline metals
Source: Nat Commun. 2017 Dec 15;8:2142. doi: 10.1038/s41467-017-02393-4 (PMC5732218; doi:10.1038/s41467-017-02393-4)
Supplement: Supplementary file 1 — Supplementary Information [file 41467_2017_2393_MOESM1_ESM.pdf]

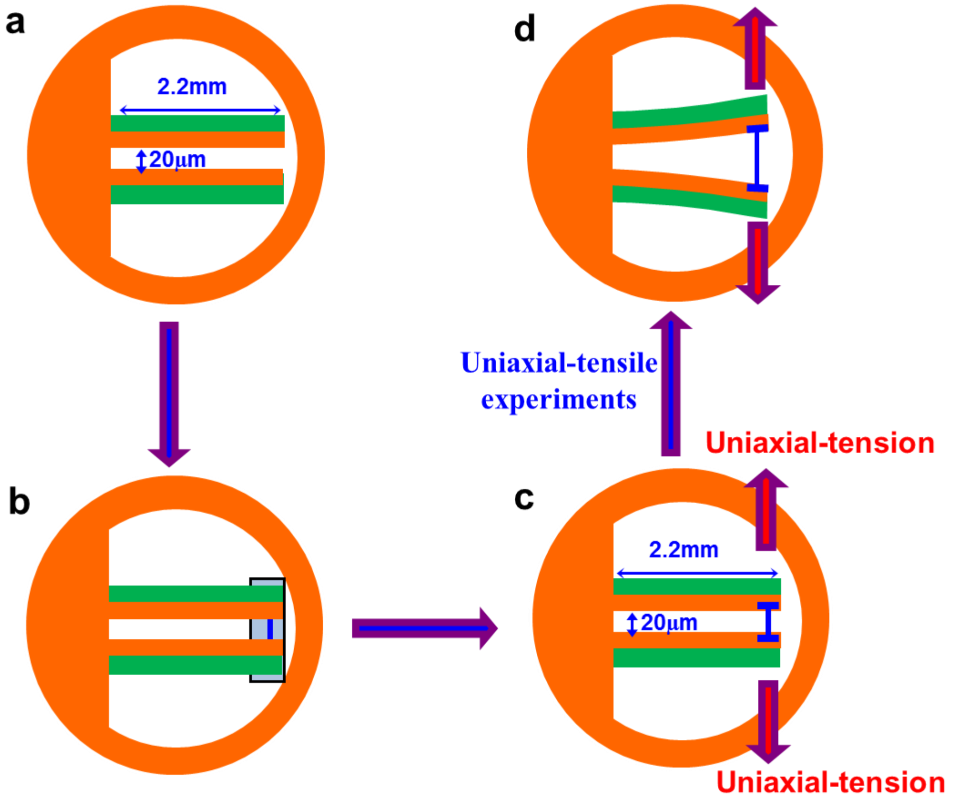

Supplementary Figure 1. Schematics of the *in situ* TEM tensile loading device. (a) Two thermal bimetallic strips were fixed on a TEM Cu-ring grid. (b) Under an optical microscope, the longitudinal direction of the bimetallic strips can be easily aligned to be perpendicular to the axial direction of the thin-film specimen. (c) Upon etching away the substrate, the bimetallic strips together with thin-film specimens were released from the substrate. (d) The double-tilt TEM tensile stage exerted uniaxial tensile forces on thin-film specimens with increasing temperature. The temperature was kept below 80 °C, which is much lower than the high melting temperature (1772 °C) of Pt. Therefore, the temperature effects on dislocations and twins should be negligible.

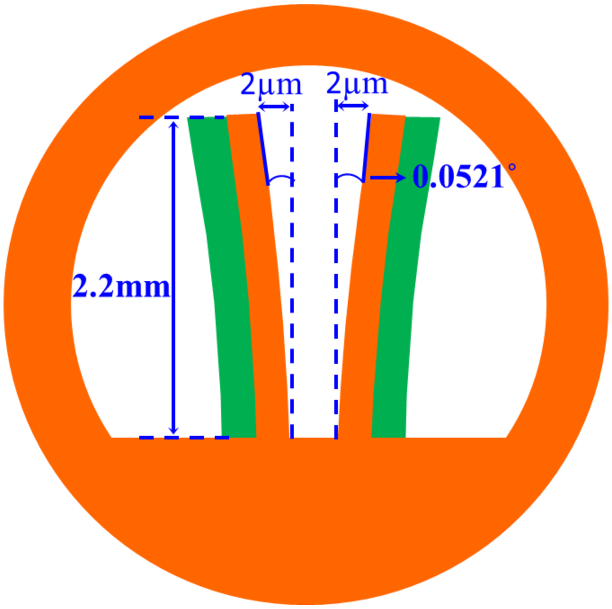

Supplementary Figure 2. Schematics showing the deflection in bimetallic strips that allows the realization of an approximate uni-axial tensile load on the Pt thin film. Schematic illustration of the bimetallic strips with length of ~2.2 mm and the maximum outward deflection of each bimetallic strip less than 2 μm (according to our in situ TEM measurement), which is only 0.091% of the length of bimetallic strips. Hence, the load in the Pt thin film is approximately uniaxial tension.

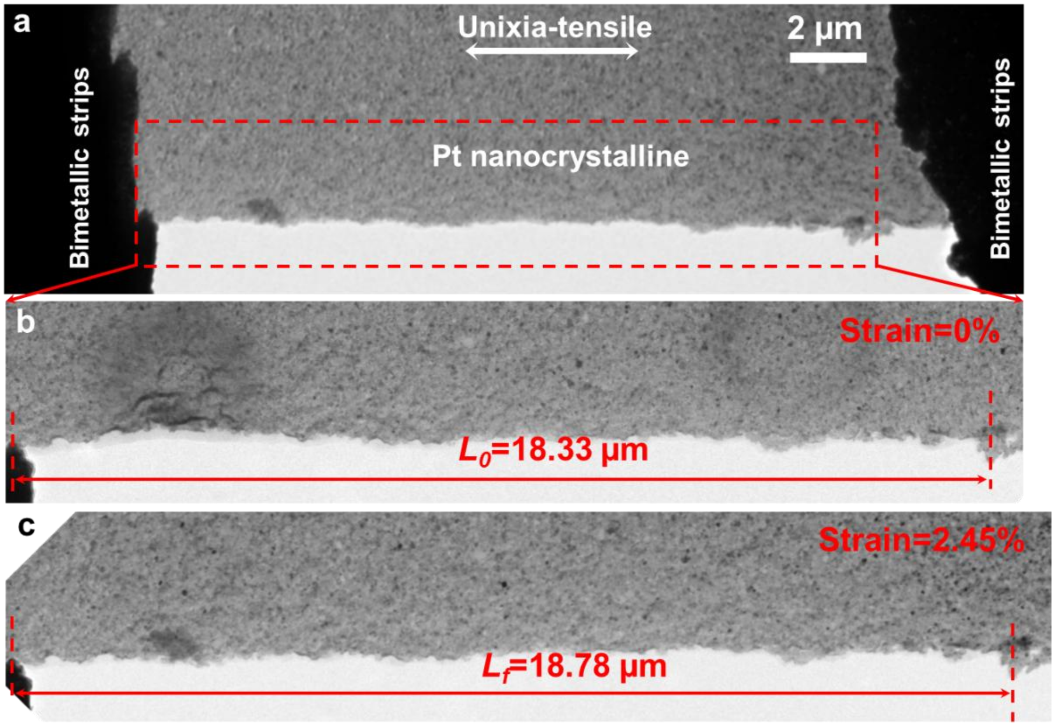

Supplementary Figure 3. The global strain estimated from the TEM images taken before and during loading. (a) A low-magnification TEM image of the Pt thin film and the bimetallic strips before the tensile loading. (b) A high-magnification TEM image corresponding to the red framed region in (a). (c) A high-magnification TEM image captured during loading. The global tensile strain was estimated as $\varepsilon = L_f - L_0/L_f$ = 2.45%.

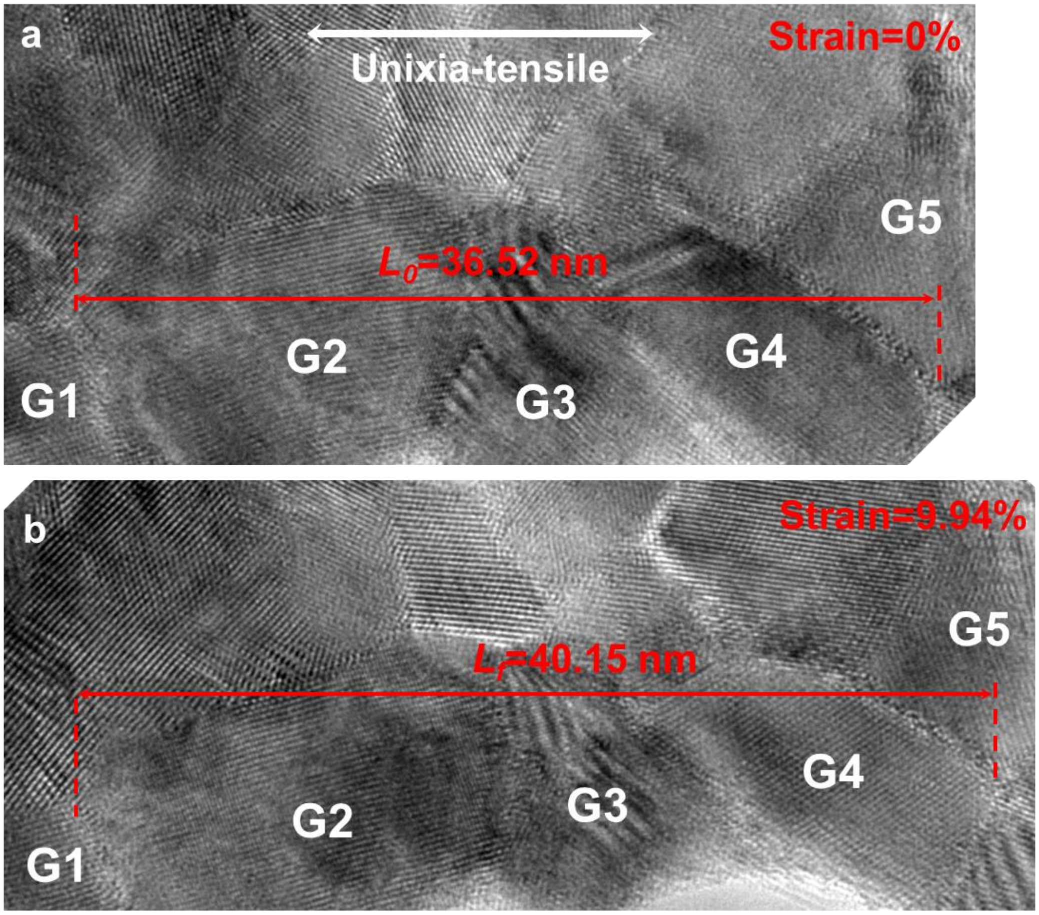

Supplementary Figure 4. Local strains estimated from the TEM images captured before and during loading. (a) A HRTEM image captured before tensile loading. (b) TEM image captured during loading. The local averaged tensile strain was estimated as $\varepsilon = L_f - L_0 / L_f$ = 9.94%.

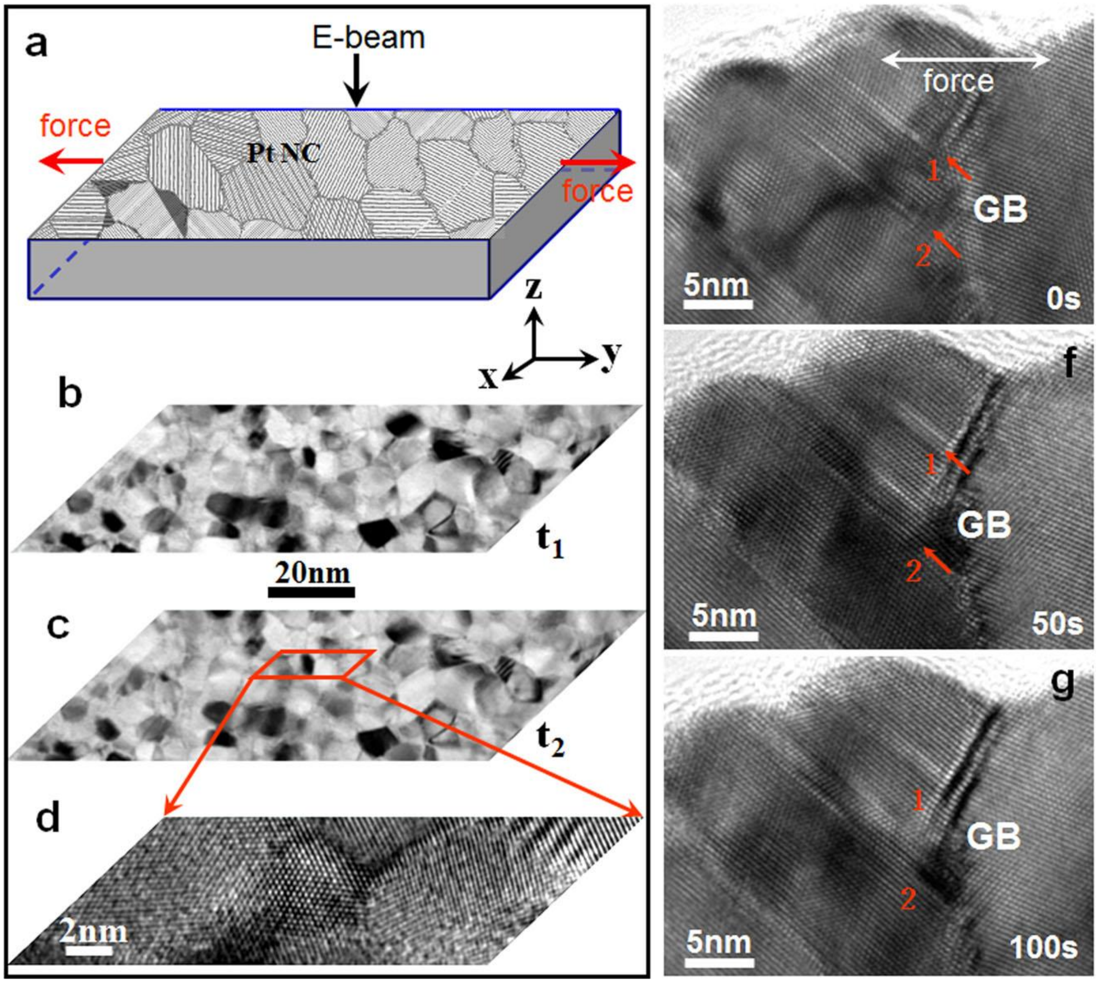

Supplementary Figure 5. (a) Schematic illustration of the loading method via a newly developed double-tilt TEM tensile stage. (b, c) Using this special TEM tensile stage, a NC Pt thin film is deformed slowly and gently while the double-tilt capability was retained. As a result, grains can be oriented appropriately, and (d) the atomic-scale deformation process can be recorded during loading. (e-g) Time series of HRTEM images acquired 50 s apart. The atomic-scale process of twin nucleation and growth was directly observed in a grain of area of ~12 nm×8.5 nm.

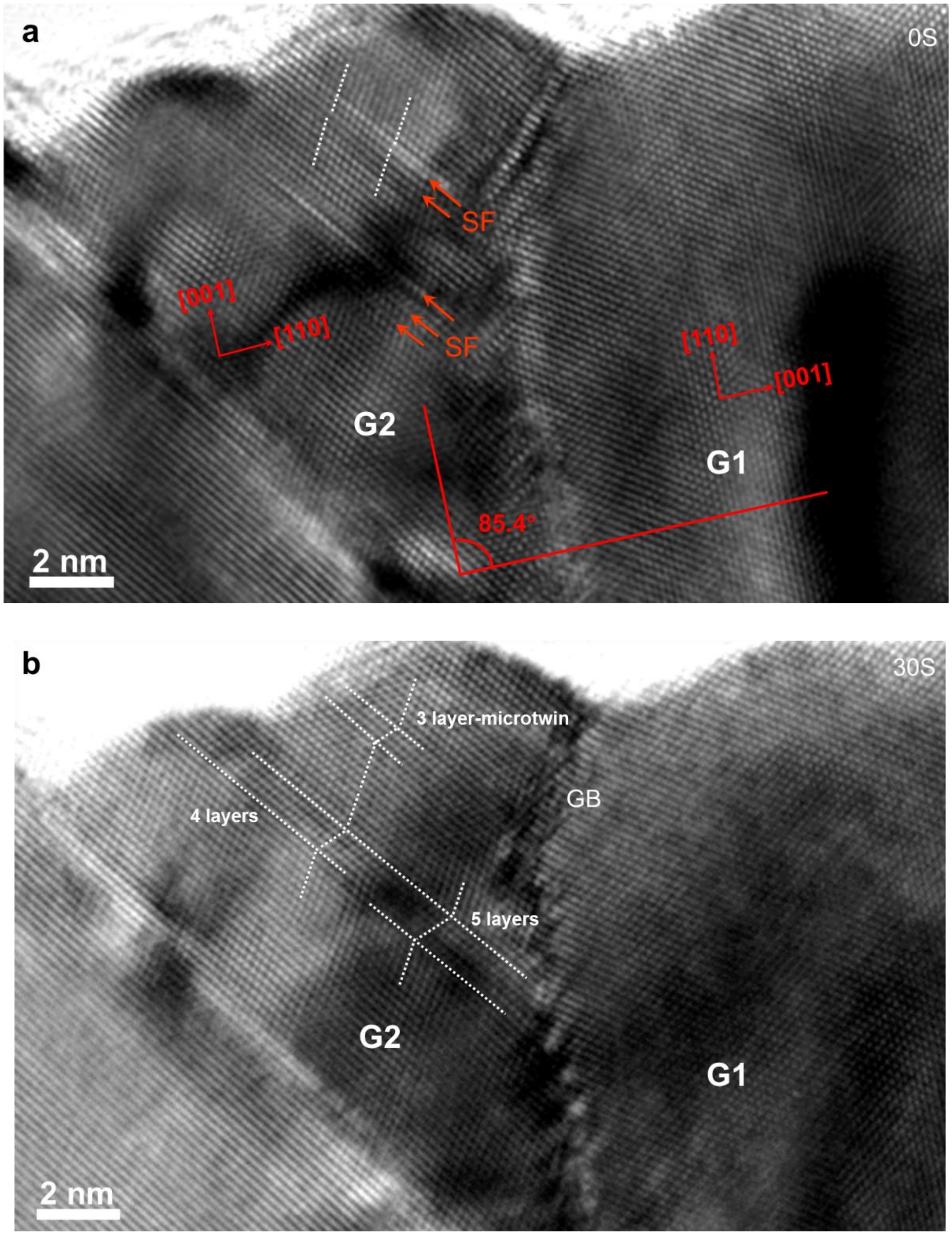

Supplementary Figure 6. Enlarged inverse fast Fourier transformed HRTEM images showing the nucleation of deformation twins via the1-3-2 twinning mode. (a) The configuration of 1-3 SFs is indicated by arrows. These SFs resulted from the partial dislocations emitted from grain boundaries (GBs). Both the G1 and G2 grains exhibit lattice contrast, indicating that the GB between G1 and G2 is of the tilt high-angle type. (b) A 3-layer twin formed via the emission of a SF between the 1-3 SFs previously formed.

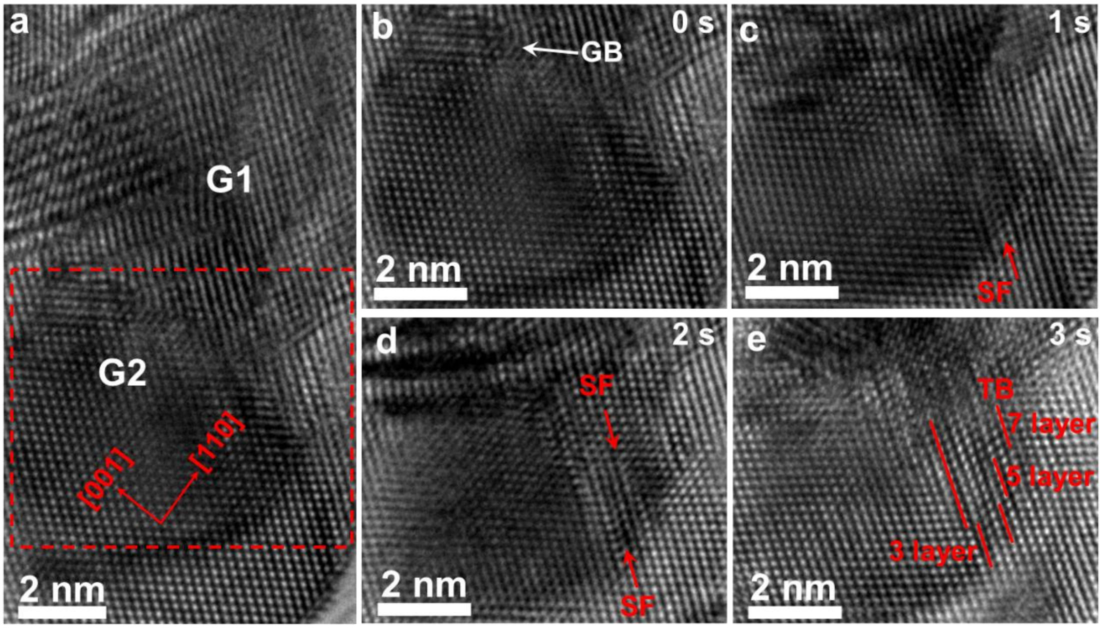

Supplementary Figure 7.An additional example showing a 3-layer twin formed via the1-3-2 twinning mode (as defined in the paper). (a) The G1 grain shows the fringe contrast while the G2 grain exhibits the lattice contrast, indicating that the GB between G1 and G2 is of the mixed tilt-twist type. (b) A perfect grain that does not contain any partial dislocation. (c) Formation of a single SF through the emission of a partial dislocation from the GB. (d) 1-3 SFs formed. (e) A 3-layer twin formed via the 1-3-2 SFs, and this twin further grew in a layer-by-layer fashion. Twin boundary (TB) steps were observed. These steps correspond to the partial dislocations emitted from the GB-TB intersection.

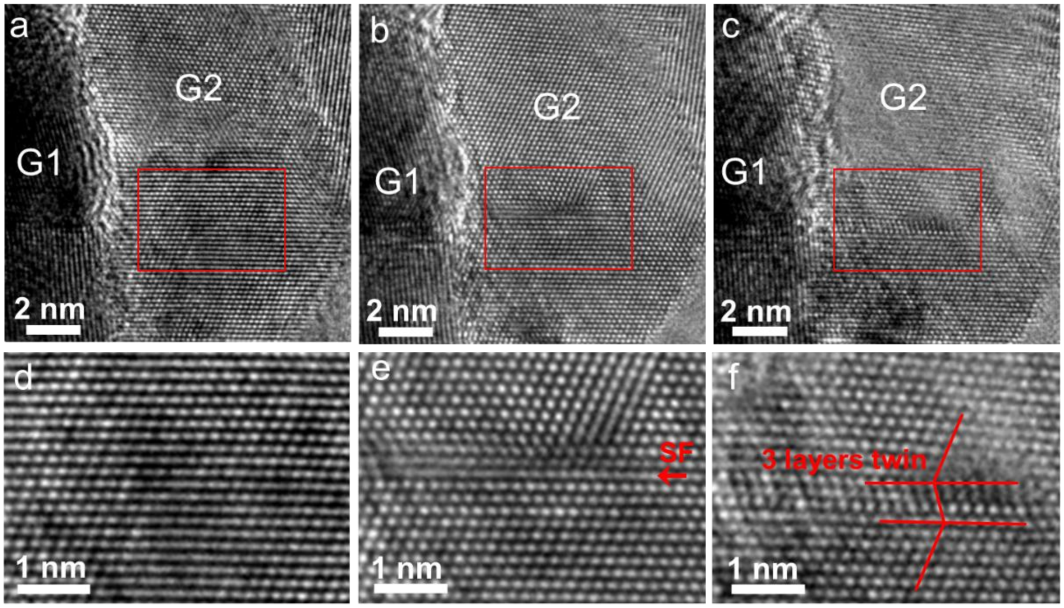

Supplementary Figure 8.Additional HRTEM examples showing the nucleation of 3-layer twins with 1-3-2 SFs (as defined in the paper). (a) A perfect grain that did not contain any partial dislocation. The G1 grain shows the fringe contrast while G1 exhibits the lattice contrast, indicating that the GB between G1 and G2 is of the mixed tilt-twist type. (b) Formation of a single SF through the emission of a partial dislocation from the GB. (c) A 3-layer twin nucleated via 1-3-2 SFs. (d-e) Enlarged HRTEM images corresponding to the boxed regions in (a-c), respectively.

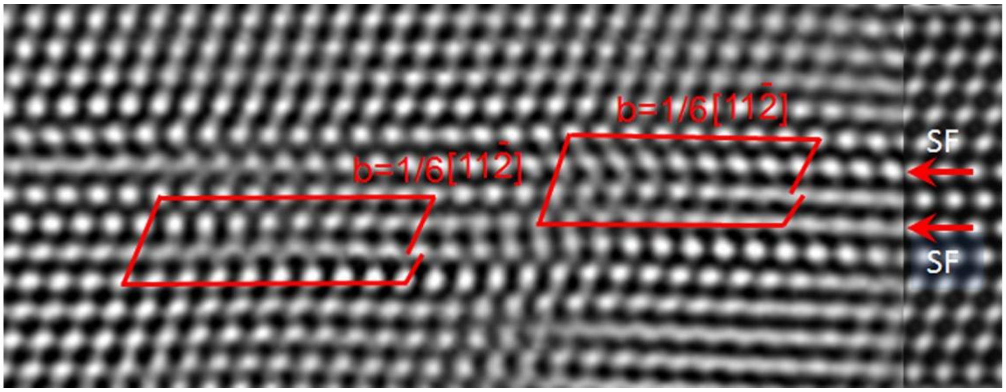

Supplementary Figure 9. An enlarged inverse fast Fourier transformed HRTEM image showing the configuration of 1-3 SFs. The Burgers circuit analysis indicates that the SFs are associated with the partial dislocations with the Burgers vector of $\frac{1}{6}[11\bar{2}]$.

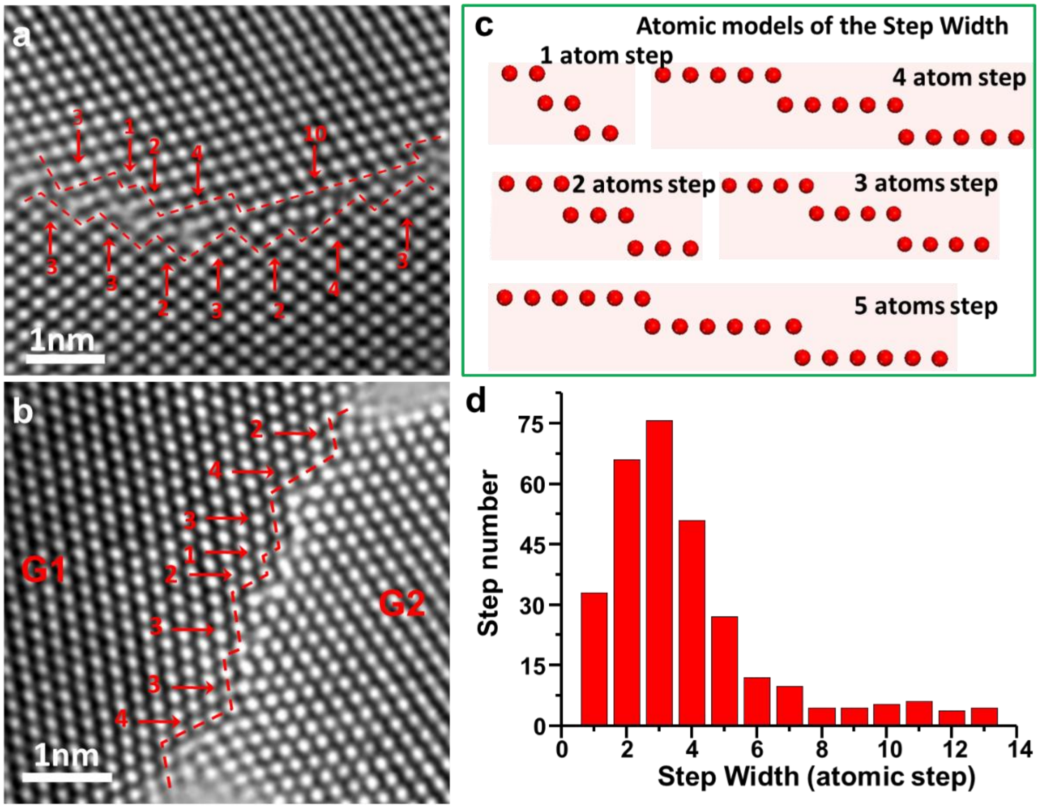

Supplementary Figure 10. Typical GB structures in grains of size between ~6-10 nm. (a, b) Cs-corrected HRTEM images showing a high density of atomic-sized steps. (c) Schematic illustration of the GB step width. (d) Histogram of the GB step widths collected from a large number of GBs in grains of size between ~6-10 nm, showing the highest density of three-atom-sized steps. These three-atom-sized steps facilitate the nucleation of 1-3 SFs.

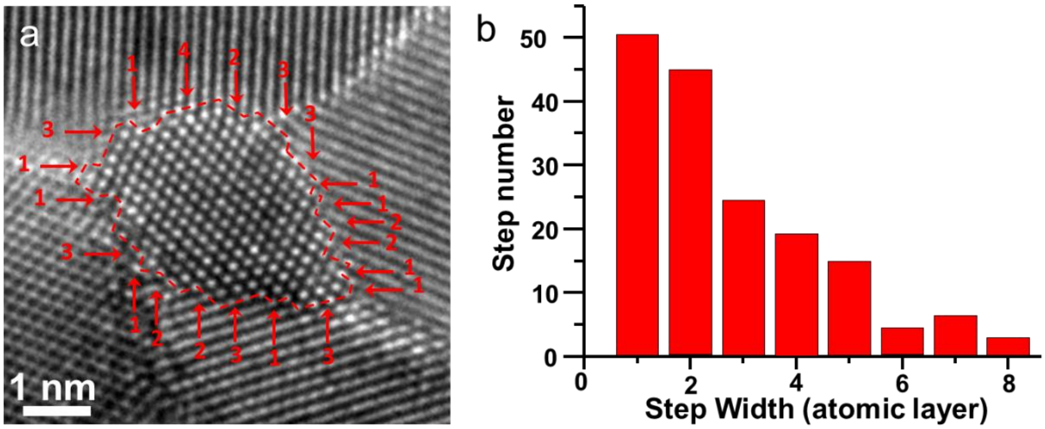

Supplementary Figure 11. Typical GB structures in grains of size less than ~6 nm. (a) Cs-corrected HRTEM images showing a high density of atomic-sized steps. (b) Histogram of the GB step widths collected from a large number of GBs in grains of size less than ~6 nm, showing the highest density of one- and two-atom-sized steps.

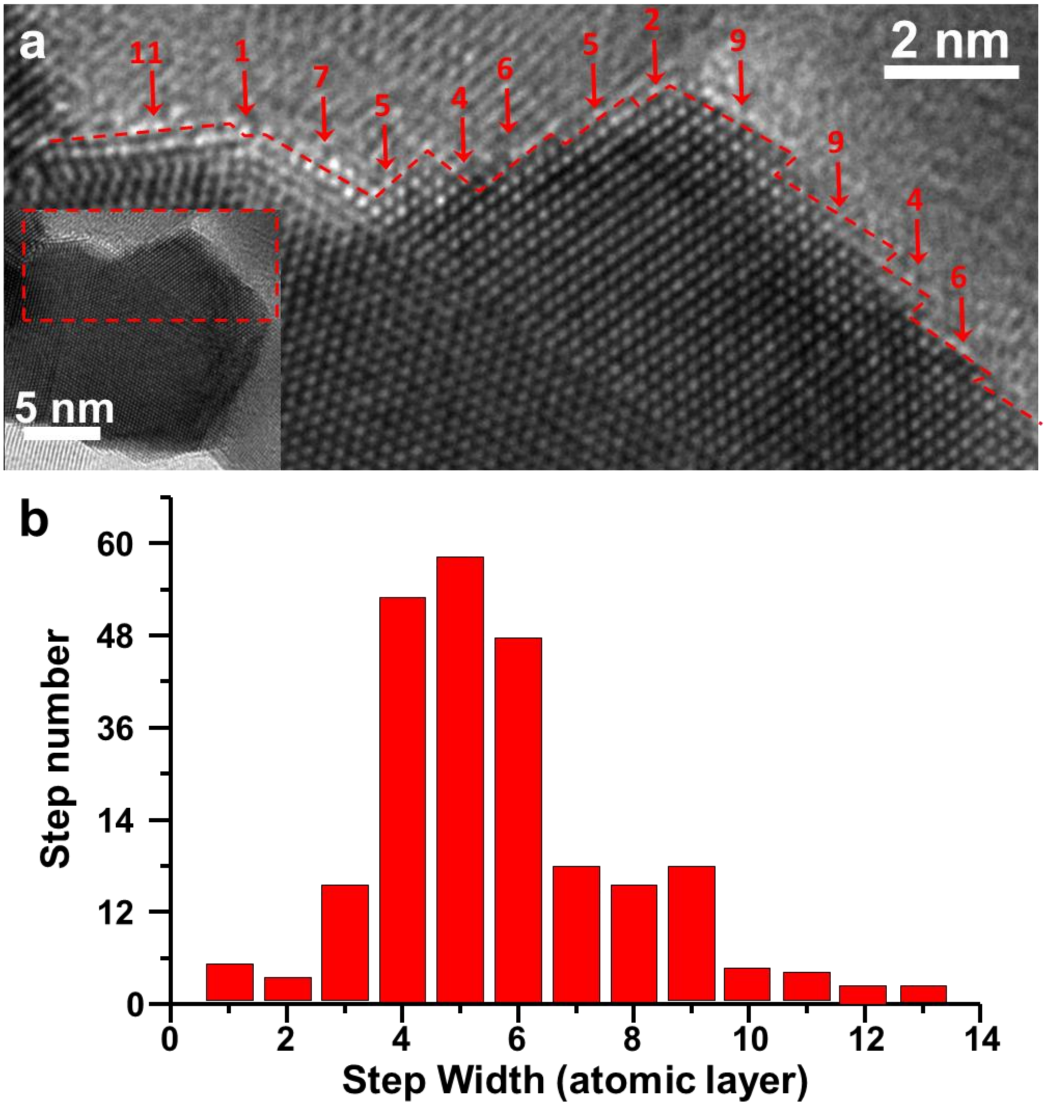

Supplementary Figure 12. Typical GB structures in grains of size larger than ~10 nm. (a) HRTEM images showing a high density of atomic-sized steps. (b) Histogram of the GB step widths collected from a large number of GBs in grains of size larger than ~10 nm, showing the highest density of five and six-atom-sized steps.

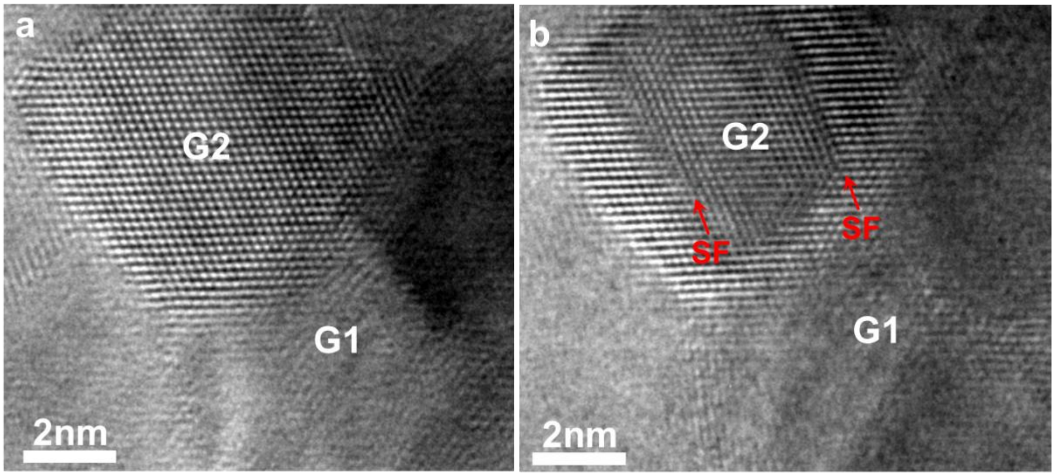

Supplementary Figure 13. HRTEM images showing the separated SFs in two grains of size of ~6-10 nm. The grain G2 shows the lattice contrast, while no fringe or lattice contrast was observed in G1. Hence, it is difficult to determine whether the GB is of tilt, twist or type. But it is likely a high-angle GB.

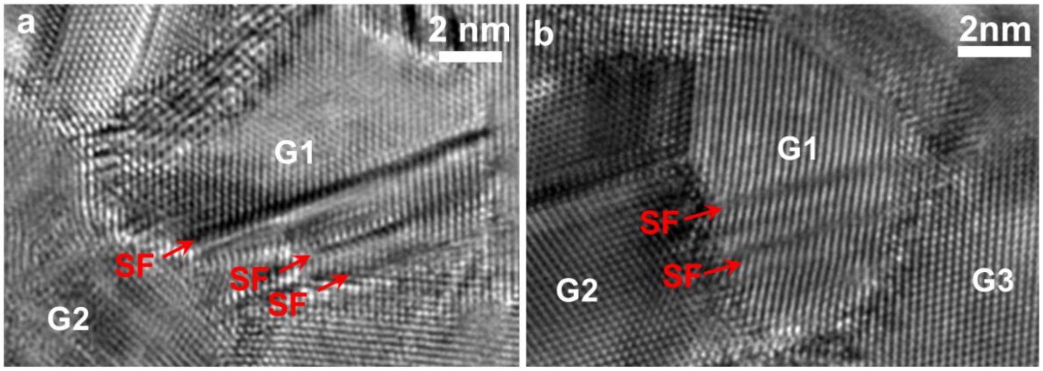

Supplementary Figure 14. HRTEM images showing the SFs separated by four or more atom layers in grains of size of ~6-10 nm. (a) The grain G1 shows the lattice contrast, while the grain G2 exhibits the fringe contrast, indicating that the GB between G1 and G2 is of mixed tilt-twist type. (b) The grain G1 exhibits the fringe contrast, while the grains G2 and G3 exhibit the lattice contrast, indicating that the GBs are of mixed tilt-twist type.

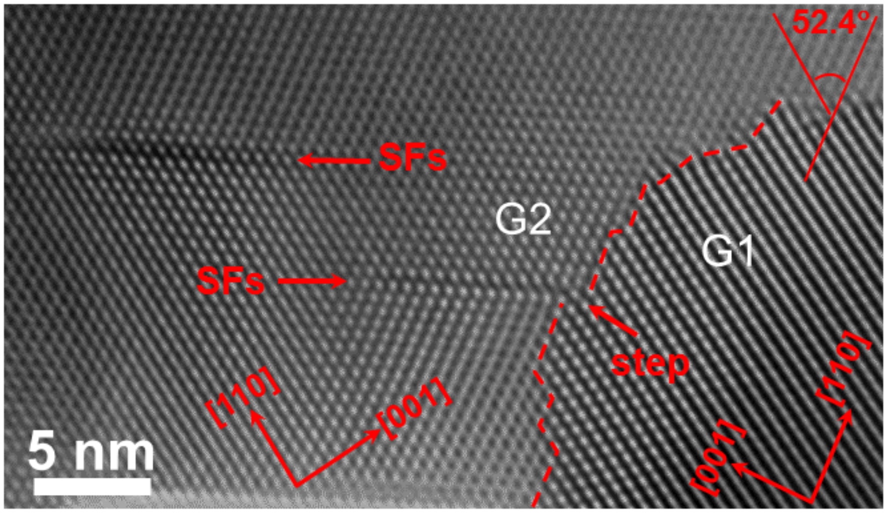

Supplementary Figure 15. Cs-corrected HRTEM images showing the partial dislocation nucleated from the GB step. Both the grains G1 and G2 exhibit the lattice contrast, indicating that the GB is of high-angle tilt type.

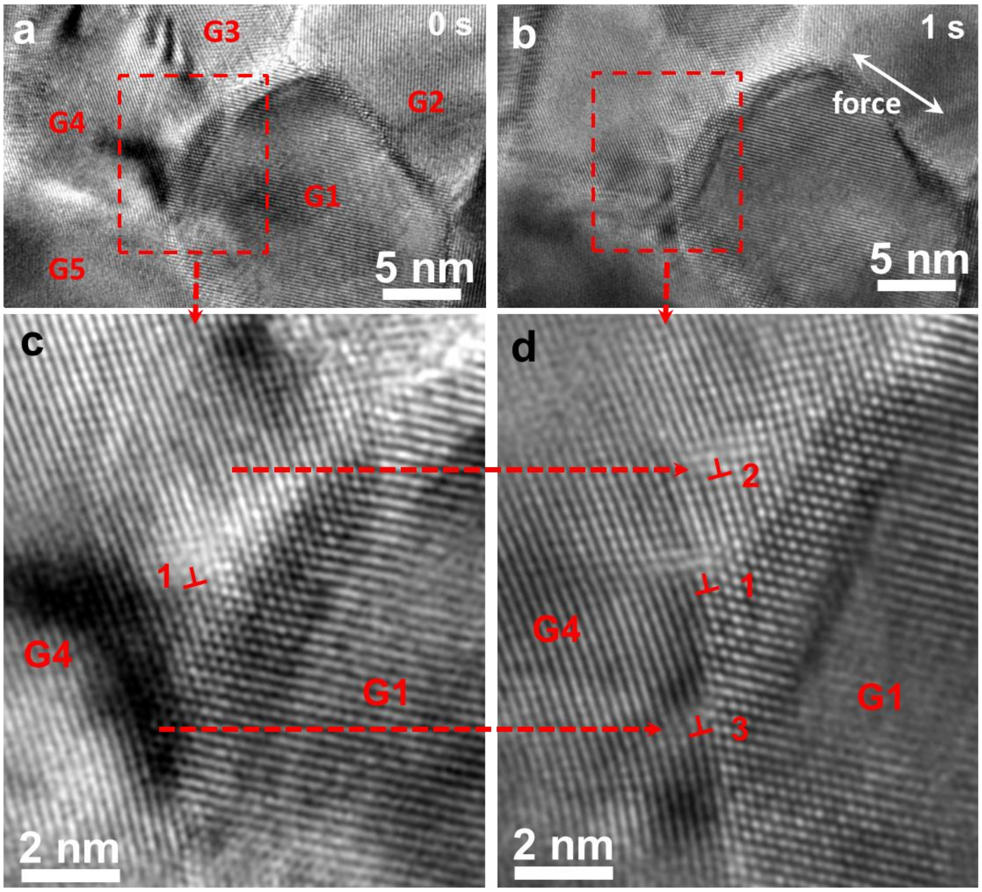

Supplementary Figure 16. HRTEM images showing the full dislocation nucleated from the GB in relatively large grains (*d* > 10 nm). (a,b) Two low-magnification TEM images captured 1 s apart. Both the grains G1 and G4 exhibit the fringe contrast, such that it is difficult to determine the GB type. (c,d) HRTEM images corresponding to the red framed region in (a) and (b), respectively. The full dislocations (marked by “2” and “3”) nucleated from the GB.

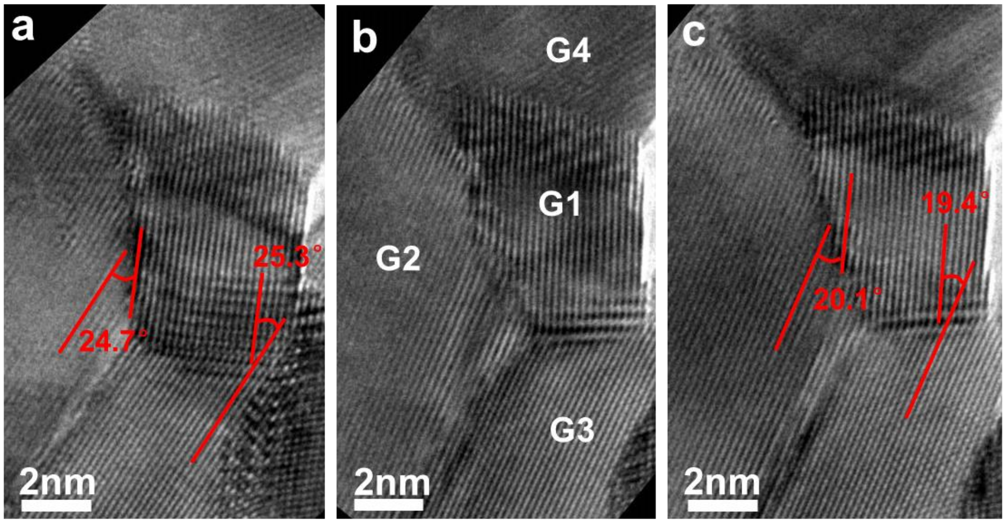

Supplementary Figure 17. In situ observation of grain rotation in small grains less than ~ 6nm. (a) The GB angles are ~24.7° and 25.3° between grains G1/G2 and G1/G3, respectively. (b,c) During loading, the misorientation angle between grains G1/G2 decreased from 24.7° to 20.1°, while that between G1/G3 from 25.3° to 19.4°. No dislocations were observed inside these small grains throughout the deformation process.

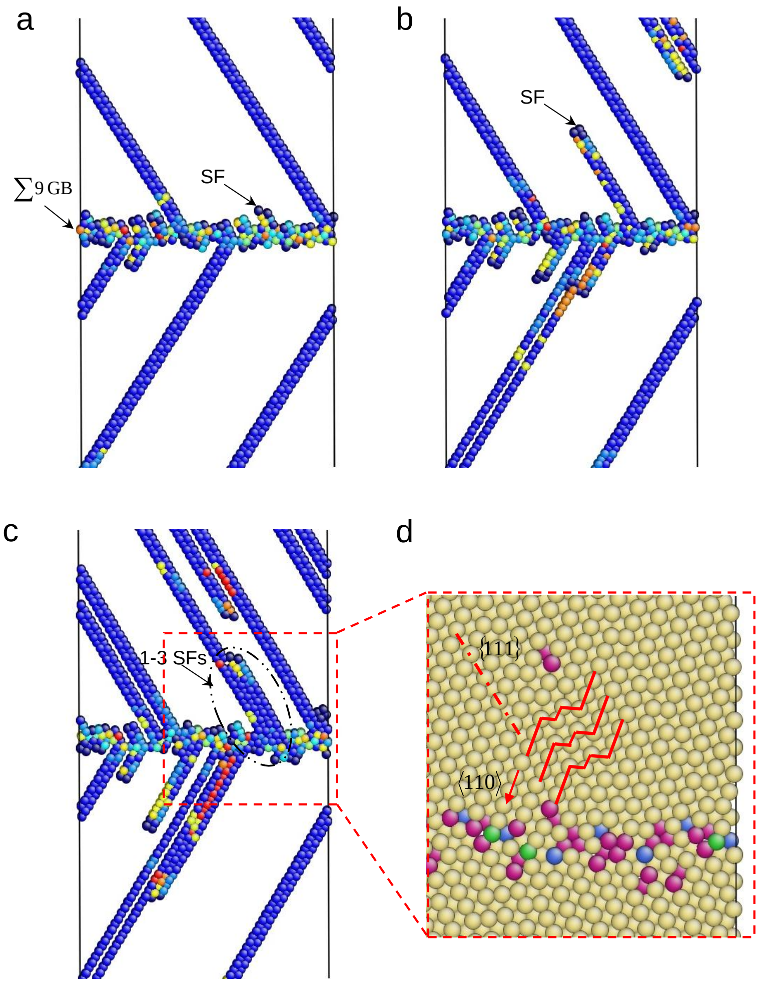

Supplementary Figure 18. MD snapshots showing the nucleation of a pair of 1-3 SFs from a Pt symmetric tilt $\Sigma 9$ GB with a number of atomic-sized steps. (a) A SF is nucleating from a step edge at the GB. (b) The incipient SF in (a) has been emitted from the GB and grown into the grain. (c) Nucleation of another SF from the GB, leading to the formation of a pair of 1-3 SFs separated by one atomic layer. The simulation supercell is viewed along the $\langle 110 \rangle$ direction. Atoms in (a-c) are colored by the central symmetry parameters, such that only the defective atoms at grain boundaries and SFs are displayed. (d) An enlarged view of the boxed region in c, showing 1-3 SFs separated by one atomic layer (i.e., 1-3 SFs). The red solid lines indicate the closed-packed $\langle 110 \rangle$ direction. Atoms are colored by the coordination number N (yellow: N=12 (a perfect FCC lattice); pink: N=11; blue: N=13; green: N=10).

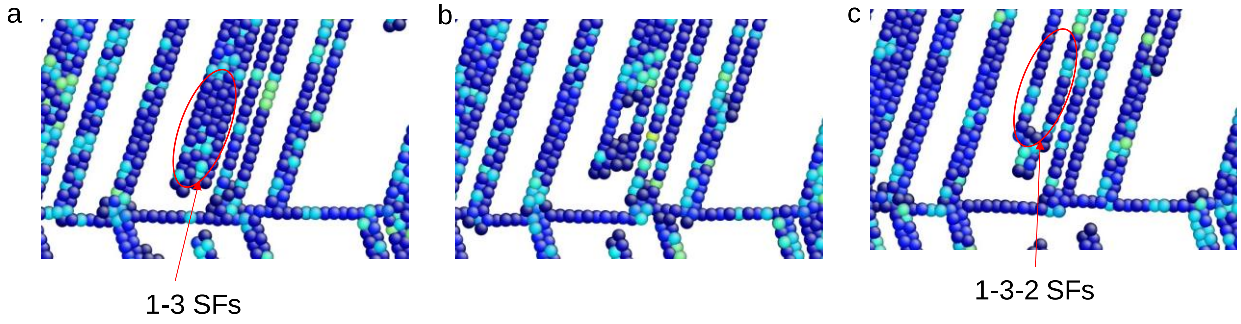

Supplementary Figure 19. MD snapshots showing the 1-3-2 twinning mode from a Pt symmetric tilt GB of $\Sigma 291$ (tilt angle $\theta = 131.5^{o}$). (a) Formation of 1-3 SFs. (b-c) Formation and expansion of a SF in between the 1-3 SFs, resulting in the 1-3-2 twining mode.

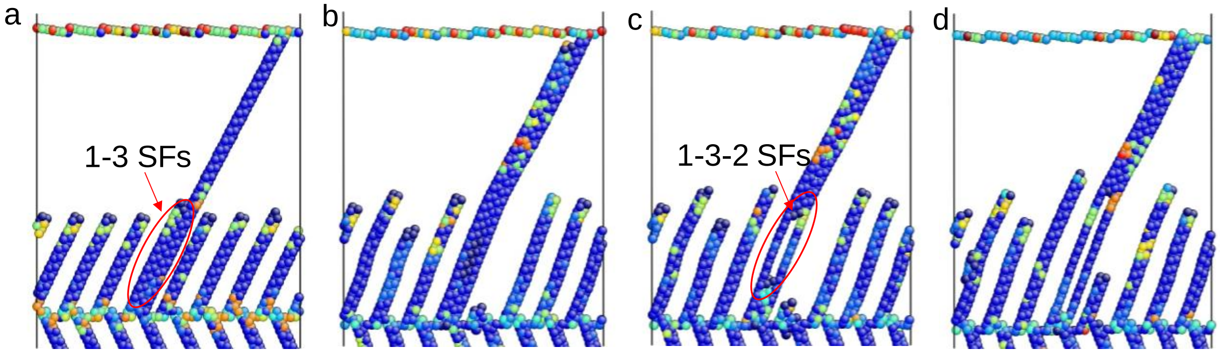

Supplementary Figure 20. MD snapshots of the 1-3-2 twinning mode from a Cu symmetric tilt GB of $\Sigma 291$ (tilt angle $\theta = 131.5^{o}$). (a-b) Formation and expansion of a SF close to an adjacent SF, leading to the formation of 1-3 SFs. (c-d) Formation and expansion of a SF in between 1-3 SFs, resulting in the 1-3-2 twining mode.

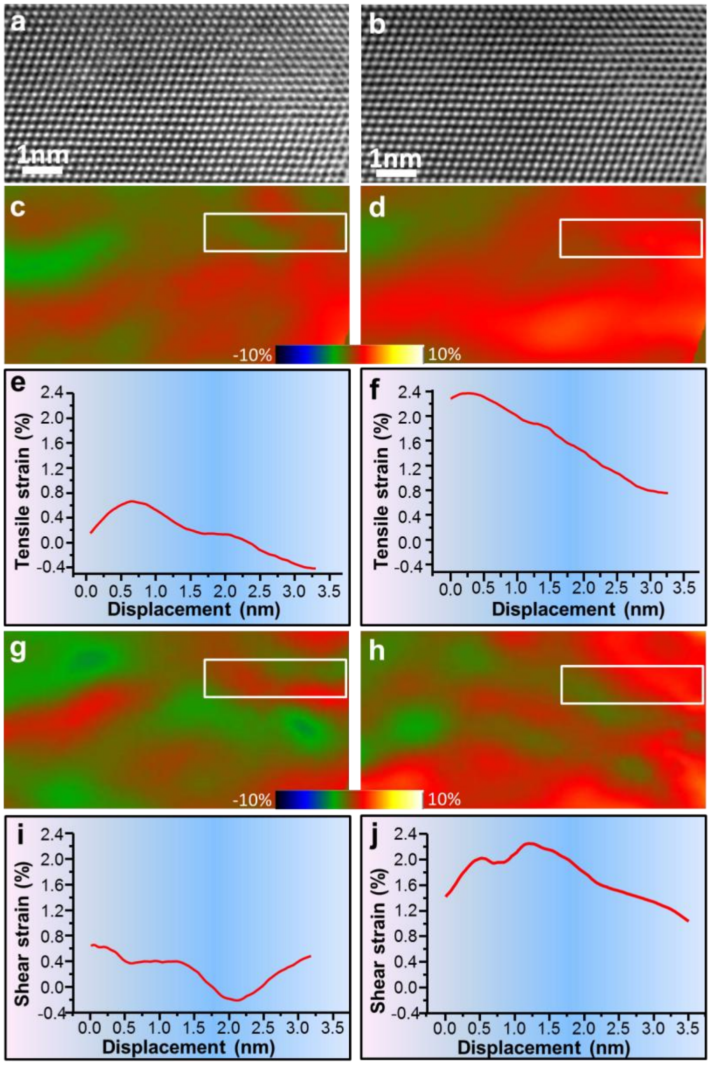

Supplementary Figure 21. Two Cs-corrected HRTEM images, acquired 0.5 s apart, showing the increasing tensile and shear strain during loading. (a) The strain is initially low. (b) With further loading, the strain increases to approach the point of partial dislocation nucleation. (c, d) Tensile strain maps (ε_{yy}) corresponding to (a, b). (e, f) Quantitative tensile strains extracted via a line-intensity scanning from the right to left in the boxed regions of (c, d). The largest tensile strain is ~2.4% for the nucleation of 1-3 SFs. (e, f) Shear strain maps (ε_{xy}) corresponding to (a, b). (e, f) Quantitative shear strains extracted via a line-intensity scanning from the right to left in the boxed regions of (c, d). The largest shear strain is ~2.2% for the nucleation of 1-3 SFs. Thus, the shear stresses for partial dislocation emission and accordingly twin nucleation are estimated to be approximately 1.44 GPa for Pt with a shear modulus of 65.4 GPa.

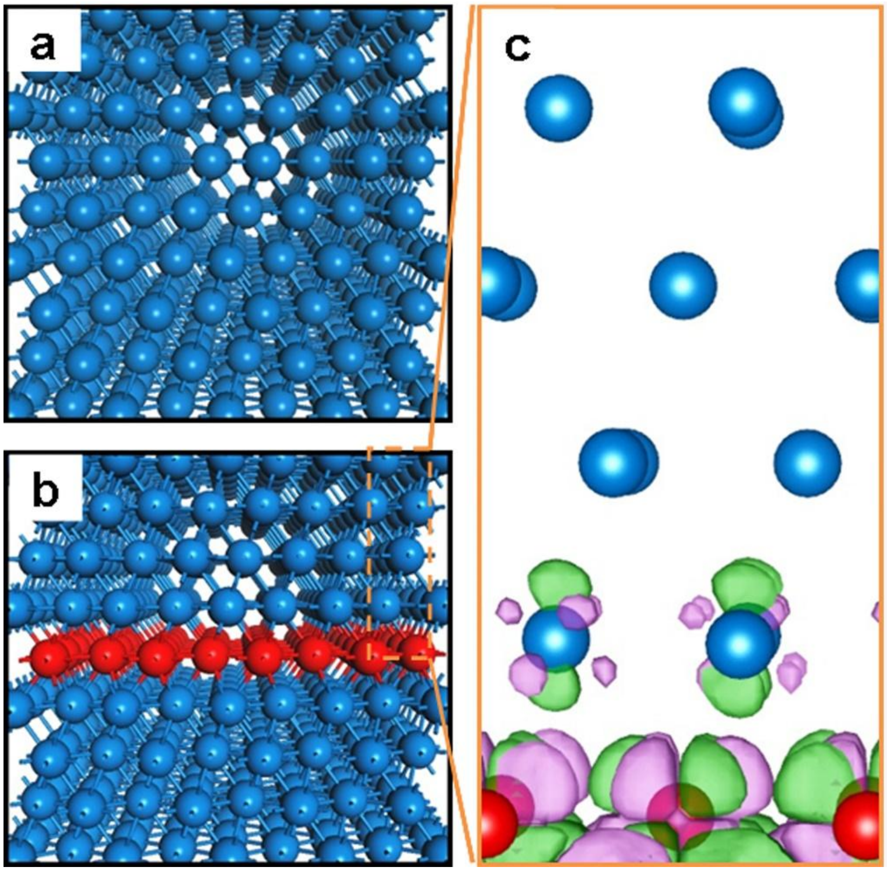

Supplementary Figure 22. Density functional theory calculation results. (a) A 3D atomic model showing the perfect FCC lattice of Pt. (b) A 3D atomic model with a single SF in an otherwise perfect FCC lattice. (c) Differences in the charge density distribution between configurations in (a) and (b); only the framed region in (b) is shown in (c). The values of iso-surface of charge density are $\pm 1.0 \times 10^{-3} \text{eÅ}^{-3}$. By comparing the charge density difference between (a) and (b), we find that the charge environment surrounding the 3rd layer in (b) is similar to that in (a).

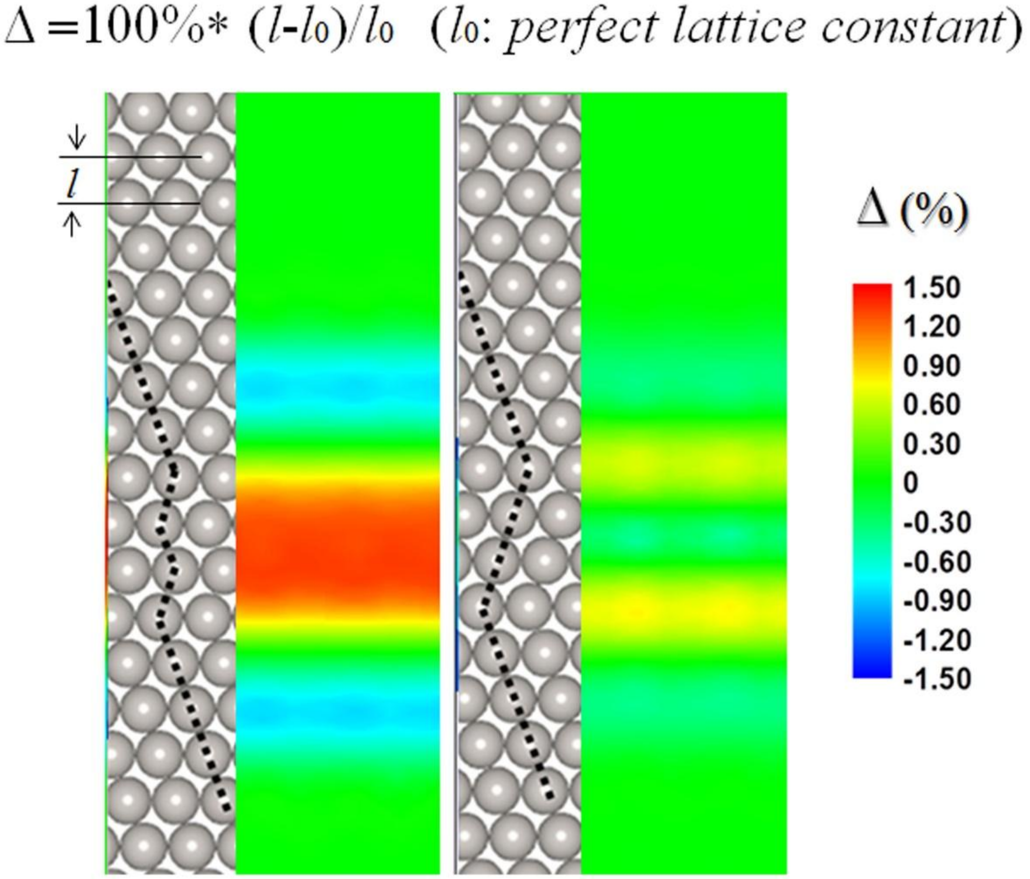

Supplementary Figure 23. Density functional theory calculation results showing the atomic structures and corresponding strain maps for Path B. Emission of the third partial dislocation between 1-3 SFs drastically lowers the local atomic-level strains.

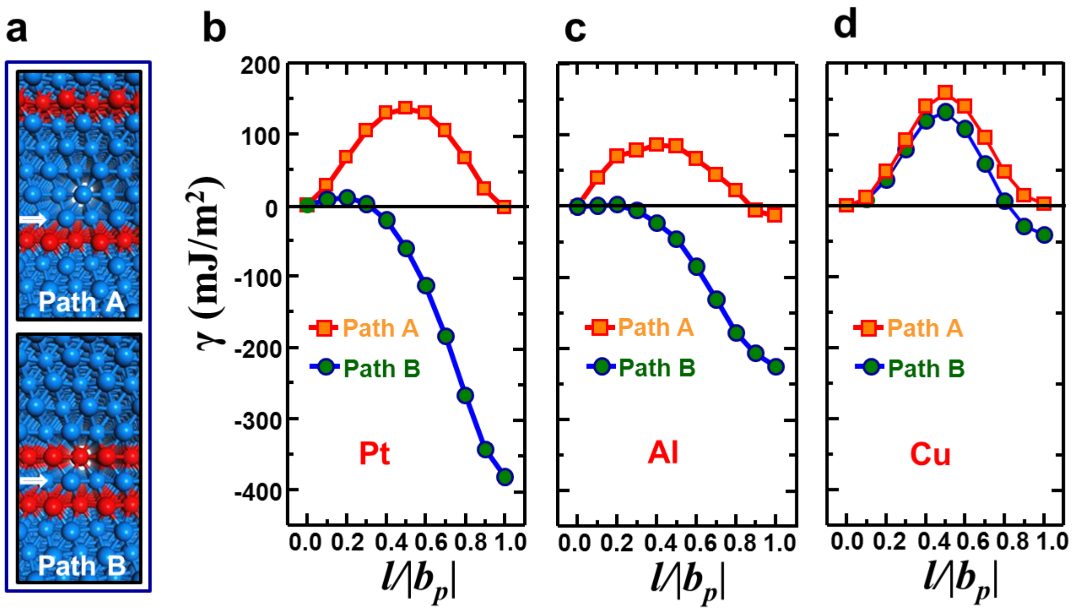

Supplementary Figure 24. Density functional theory (DFT) calculation results showing the comparison of twinning energy landscapes for paths A and B for Pt, Al and Cu. (a) Initial atomic structures for paths A (above) and B (below), where the red atomic planes indicate the SF layers. (b) DFT-calculated twinning energy landscapes of Pt along paths A and B. The energy barrier for path A (i.e., SFs form on consecutive atomic layers) is much higher than that for path B (i.e., a SF forms between 1-3 SFs). (c) Same as (b) except for Al. Similar to Pt, the energy barrier for path A is much higher than that for path B for Al. (d) Same as (b) except for Cu. In contrast to Pt and Al, the energy barriers are similar for paths A and B in Cu.

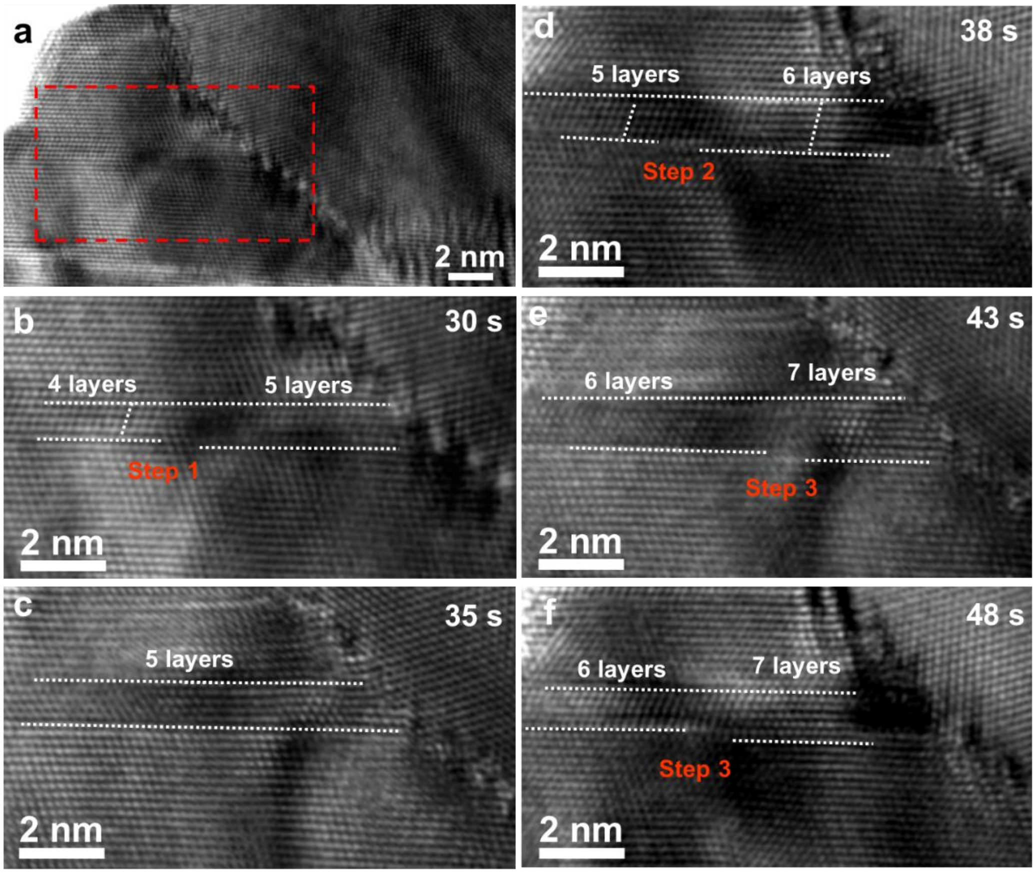

Supplementary Figure 25. *In situ* observation of TB migration in a layer-by-layer fashion. (a) HRTEM images corresponding to Supplementary Figure 6b. (b-f) Time series of enlarged HRTEMs corresponding to the red framed region of (a); these images show the twin thickening process during loading. (b) A twin-boundary (TB) step (marked as “Step 1”) of height of one atomic layer. (c) “Step 1” moves to the left, causing the migration of TB and the formation of a 5-layer twin. (d) With further loading, “step 2” in the TB was captured, and gliding of “step 2” causes the thickening of a 5-layer twin into a 6-layer twin. (e,f) Similar to (d), a new “step 3” at the TB was captured, and this step appeared to nucleate from the GB-TB intersection.
